# Food Preservatives and Appendicitis

**Published:** 1906-11

**Authors:** H. H. Langdon

**Affiliations:** 427 West 22d Street, New York


### CORRESPONDENCE.

### Food Preservatives and Appendicitis.

**Mr. Langdon, food expert, gives hs views concerning boric acid as a preservative.—Thinks it harmless, and the most innocent antiseptic extant.—Coproliths and appendicitis.—Constipation, diarrhea and other digestive disturbances prime factors in developing appendicitis.**

Editor Buffalo Medical Journal.

Sir: I have read numerous articles on the Pure Food Question and the evil effects of coloring matter and preservatives on the human system, but not until recently have I perused articles written by physicians who claim that boric acid and boron compounds, which are used quite extensively for preserving food, are the cause of appendicitis. An article appeared in the "New York Medical Journal" April 17, 1906, and in "Truth," of Buffalo, June 30, 1906, stating that boric acid was the cause of appendicitis.

If such statements were true, however, the English Nation would be wiped out of existence. They have consumed .foods preserved with borax for decades, and if food preserved with Boron Compounds was dangerous to health, the entire medical fraternity would have learned of it years ago.

I have had a great deal of experience with boric acid and have always found it a soothing, cooling, healing, sedative agent. The action of boric acid on the cuticle and mucous membrane is to allay inflammation, not to cause it. It is recognised as the most innocent antiseptic extant. It is an antiseptic which never irritates nor inflames and thus enables a natural healing process to take place without interruption. Its action on the organic tissues is seen by the blood. Concentrated boric acid mixed thoroughly with fresh blood only delays and cannot prevent coagulation.

In spite of all that has been said against boric acid, it is clear that its action on albuminous bodies has no analogy with any other acid except carbonic acid gas. It has been stated that weak or diseased kidneys could not eliminate boric acid. It is a fact, however, that it forms remedies of great value in kidney diseases. If the vermiform appendix were inflamed, boric acid would have a tendency to allay the inflammation instead of exciting it. Solutions of boric acid have been used in every cavity of the human system with beneficial instead of detrimental results.

That cases of appendicitis are more numerous now than they were years ago cannot be denied. Years ago, however, such cases were diagnosed differently. In the census year of 1890 there are no records of any appendicitis cases. In 1900, five thousand one hundred and eleven cases were reported. There is no doubt that a few cases are caused by foreigns bodies entering the appendix. Coproliths are found much more frequently, however, than foreign bodies.

Bryant, in his paper, published in the "Annals of Surgery," February, 1903, states, "I found in one hundred and twenty-four cases, abnormal matter in 70 per cent. of the males and 55 per cent. of the females." Renvers, in the "Deutsche Medicinsche Wochenschrift," 1891, found in four hundred and fifty-nine autopsies, one hundred and seventy-nine coproliths and about sixteen foreign bodies.

We do not as yet understand the functions of the appendix. Without doubt almost every case of inflammation in the iliac region can be traced to a diseased appendix. Fecal matter is forced into the appendix, which is so constructed that it cannot drain itself, hence causes inflammation. The veriform appendix being a weak organ is unable to protect itself.

Constipation would have a tendency to interfere with the supply of blood by direct pressure on the single artery which supplies the blood.

A great many cases can, no doubt, be attributed to our bad habits of eating too much and masticating our food too little, which causes constipation, dyspepsia and general derangement of the functions. The hurrying restless lives we lead certainly interfere with the normal working of our digestive organs. I firmly believe that indigestion, constipation, diarrhea and other digestive disturbances are the prime factors which favor the development of appendicitis.

H. H. Langdon.

427 West 22d Street,
New York, September 27, 1906.

---

Obstruction of the common bile duct.—John F. Erdmann, after discussing the causes and symptoms of bile-duct obstruction, says that when all medical aid has been given without results, or if the symptoms are so urgent as to demand immediate surgical interference, the condition narrows itself to a question of surgical technique and after-treatment. The writer in exposing the duct, makes the incision over the right rectus, splitting the fibres in the middle and internal, or middle and external thirds, for a distance sufficiently long to explore the duct. If the stone is located or adhesions demand it, the incision is rapidly enlarged to give ample working room. After the stone is removed, a fish-tail rubber tube, with from one to three openings, and about ten or twelve inches long, is passed into the duct in its proximal direction for its lower three-quarters of an inch, sufficiently to bury the apertures only. The tube is held in place by a suture of chromic catgut. The history of an interesting case concludes the paper.—*Medical Record,* September 22, 1906.

---

"I always thought," remarked an English judge, "that a parasol and a sunshade were the same."

"No," replied the witness on the stand; "a sunshade is to keep the sun off; a parasol is to flirt with."

Necessarily.—Dinglebats: The oculist charged you $5 for taking a grain of sand out of your eye? That's pretty steep, isn't it?

Himpsley: I thought so, till I looked over his bill. It was for "removing foreign substance from the cornea," and, of course, that costs more.—*Chicago Tribune.*

